# Selenium and risk of bladder cancer

**DOI:** 10.1186/1897-4287-10-S3-A23

**Published:** 2012-04-20

**Authors:** E Złowocka-Perłowska, M Słojewski, A Sikorski, G Sukiennicki, J Lubiński

**Affiliations:** 1 International Hereditary Cancer Center, Department of Genetics and Pathology, Szczecin, Poland; 2 Urology Clin SPSK2, Szczecin, Poland; 3 Read-Gene SA and Pomeranian Medical University Szczecin, Poland

## 

Urothelial bladder cancer is the second most common tumor of the genitourinary tract. In Poland 3,500 new cases of bladder cancer are diagnosed annually. Bladder cancer develops more predominantly in males and increases significantly in incidence between the ages of 60 and 70 years in the life. The risk factors for developing bladder cancer are: cigarette smoking, exposure to aromatic amines, polycyclic aromatic hydrocarbons and infection with Schistosoma haematobium. There is evidence that some bladder cancers are a result of a genetic predisposition to disease although up to now the genes of the high penetrance to bladder cancer have not been found. This situation calls for the development of innovative therapies based on environmental features for bladder cancer prevention and control. One potential novel approach is through diet. Some studies indicate that diet modifications may prevent incidence of bladder cancer or reduce the risk of recurrence or progression. Several epidemiologic evidence suggest that selenium concentration is inversely related to risk of bladder cancer. A Finnish cohort study found a protective effect for men but not for women. An American nested case-control study observed a statistically significant inverse association between selenium toenail concentration and risk of bladder cancer in women but not in men. Some data are conflicting and did not find this association.

To validate the association between selenium concentrations and bladder cancer risk in Polish population we have investigated 37 cases of bladder cancer and 37 controls. In this analysis, a single control was selected for each case, based on sex, year of birth (+/- 2 years) and smoking status (+/- 20% pack-years). We conclude that high selenium concentration shows the tendency to decrease the risk of bladder cancer in the Polish population (OR 2.1; 95%CI 0.5-7.9; p=0.33) but further investigations are needed to achieve statistically significant result.

